# The Effect of Post-Resistance Exercise Amino Acids on Plasma MCP-1 and CCR2 Expression

**DOI:** 10.3390/nu8070409

**Published:** 2016-07-02

**Authors:** Adam J. Wells, Jay R. Hoffman, Adam R. Jajtner, Alyssa N. Varanoske, David D. Church, Adam M. Gonzalez, Jeremy R. Townsend, Carleigh H. Boone, Kayla M. Baker, Kyle S. Beyer, Gerald T. Mangine, Leonardo P. Oliveira, David H. Fukuda, Jeffrey R. Stout

**Affiliations:** 1 Institute of Exercise Physiology and Wellness, Educational and Human Sciences, University of Central Florida, 12494 University Blvd, Orlando, FL 32816, USA; adam.wells@ucf.edu (A.J.W.); adam.jajtner@knights.ucf.edu (A.R.J.); alyssa.varanoske@ucf.edu (A.N.V.); david.church@ucf.edu (D.D.C.); carliegh.boone@ucf.edu (C.H.B.); kayla.baker@ucf.edu (K.M.B.); kyle.beyer@ucf.edu (K.S.B.); leonardo.oliveira@ucf.edu (L.P.O.); david.fukuda@ucf.edu (D.H.F.); jeffrey.stout@ucf.edu (J.R.S.); 2 Department of Health Professions, Hofstra University, Hempstead, NY 11549, USA; adam.m.gonzalez@hofstra.edu; 3 Department of Kinesiology, Lipscomb University, Nashville, TN 37204, USA; jeremy.townsend@lipscomb.edu; 4 Department of Exercise Science and Sport Management, Kennesaw State University, Kennesaw, GA 30144, USA; gmangine@kennesaw.edu

**Keywords:** inflammation, monocyte chemoattractant protein 1 (MCP-1), C-C chemokine receptor 2 (CCR2), macrophage-1 antigen (cluster of differentiation 11b (CD11b))

## Abstract

The recruitment and infiltration of classical monocytes into damaged muscle is critical for optimal tissue remodeling. This study examined the effects of an amino acid supplement on classical monocyte recruitment following an acute bout of lower body resistance exercise. Ten resistance-trained men (24.7 ± 3.4 years; 90.1 ± 11.3 kg; 176.0 ± 4.9 cm) ingested supplement (SUPP) or placebo (PL) immediately post-exercise in a randomized, cross-over design. Blood samples were obtained at baseline (BL), immediately (IP), 30-min (30P), 1-h (1H), 2-h (2H), and 5-h (5H) post-exercise to assess plasma concentrations of monocyte chemoattractant protein 1 (MCP-1), myoglobin, cortisol and insulin concentrations; and expressions of C-C chemokine receptor-2 (CCR2), and macrophage-1 antigen (CD11b) on classical monocytes. Magnitude-based inferences were used to provide inferences on the true effects of SUPP compared to PL. Changes in myoglobin, cortisol, and insulin concentrations were similar between treatments. Compared to PL, plasma MCP-1 was “very likely greater” (98.1% likelihood effect) in SUPP at 2H. CCR2 expression was “likely greater” at IP (84.9% likelihood effect), “likely greater” at 1H (87.7% likelihood effect), “very likely greater” at 2H (97.0% likelihood effect), and “likely greater” at 5H (90.1% likelihood effect) in SUPP, compared to PL. Ingestion of SUPP did not influence CD11b expression. Ingestion of an amino acid supplement immediately post-exercise appears to help maintain plasma MCP-1 concentrations and augment CCR2 expression in resistance trained men.

## 1. Introduction

Resistance exercise of sufficient intensity can result in localized damage to skeletal muscle tissue. The resolution of tissue homeostasis following damaging exercise is mediated to a large extent by cells of the innate immune system. In particular, a population of non-tissue resident macrophages appear to be key modulators of the regenerative process [1]. Depending on their polar state, these cells are reported to execute a number of processes fundamental to recovery, including the phagocytosis of opsonized cellular debris [2], and the secretion of mitogenic factors that stimulate both the proliferation and differentiation of myogenic precursor cells [3]. Under conditions of homeostasis, skeletal muscle tissue is virtually devoid of these cells. However, in response to injury, these cells begin to accumulate, and remain present within the regenerating areas throughout the recovery process [4].

Non-resident macrophages arise from the in situ differentiation of CD14^++^CD16^−^ classical monocytes, which are recruited from the circulation to the site of muscle damage [5]. Induction of muscle damage in conjunction with simultaneous depletion of CD14^++^CD16^−^ monocytes has been shown to lead to diminished macrophage infiltration, resulting in impaired muscle regeneration and a subsequent deficit in muscle fiber size [6]. Comparable impairments in recovery have been observed following abrogation of the chemotactic protein monocyte chemoattractant protein 1 (MCP-1), its counter receptor C-C chemokine receptor 2 (CCR2), and adhesion molecule macrophage-1 antigen (cluster of differentiation 11b (CD11b)) [6,7,8]. Consequently, monocyte recruitment via MCP-1 and CCR2, and endothelial adhesion via CD11b appear to be critical steps in the resolution of skeletal muscle homeostasis following myofibrillar damage.

Skeletal muscle demonstrates the ability to adapt following an initial bout of damaging exercise, such that the muscle becomes resistant to damage pursuant to subsequent bouts. This phenomenon is known as the repeated bout effect (RBE). Previously, the RBE was thought to be the result of an attenuated inflammatory response following successive bouts of damaging exercise [9]. However, more recent evidence suggests that the adaptive response to damaging exercise is facilitated by an enhancement of the inflammatory response, mediated through the augmentation of the non-resident macrophage pool in response to a robust elevation in MCP-1 [10]. Interestingly, these events are reported to coincide with a significant reduction in delayed onset muscle soreness [10], suggesting that augmentation of the monocyte response via MCP-1 signaling may lead to enhanced recovery.

Recent evidence suggests that innate immune cell function is subject to regulation by mammalian/mechanistic target of rapamycin (mTOR) signaling [11]. While there is currently no evidence to suggest that mTOR signaling is linked to monocyte CCR2 or CD11b expression, mTOR does appear to be able to modulate MCP-1 secretion by macrophages [12], and the expression of chemotactic receptors in a number of myeloid and lymphoid cells [13]. Overwhelming evidence shows that the ingestion of amino acids following damaging exercise is a potent means of enhancing recovery via mTOR dependent mechanisms [14,15]. Nevertheless, the effect of exogenous amino acids on MCP-1, CCR2 and/or monocyte CD11b expression, to the best of our knowledge has not been addressed. Therefore, the purpose of this study was to examine the effect of an amino acid supplement on plasma MCP-1 concentration in addition to monocyte CCR2 and CD11b expression following an acute bout of high-volume, moderate intensity resistance exercise in experienced, resistance-trained men. The effect of the supplement on changes in percent CD14^+^ cells relative to all leukocytes and percent CD14^++^CD16^−^ monocytes relative to all monocytes (classical, non-classical and intermediate) was also addressed.

## 2. Experimental Section

### 2.1. Participants

Ten resistance-trained men (24.7 ± 3.4 years; 90.1 ± 11.3 kg; 176.0 ± 4.9 cm; 14.1 ± 6.1% body fat) were recruited to participate in this randomized, cross-over design research study. Using the procedures described by Gravettier and Wallnau [16] for estimating samples sizes for repeated measures designs, a sample size of *n* = 10 produced a statistical power (1-β) of 0.99, based upon changes in plasma MCP-1 reported by Crystal et al. [17] for control subjects. Strict recruitment criteria were implemented to increase homogeneity of the sample. Inclusion criteria required participants to be between the ages of 18 and 35 years, with a minimum of one year of resistance training experience, and the ability to squat a weight equivalent to their body mass (confirmed during 1-RM testing). Participants had 6.7 ± 4.6 years of resistance training experience with an average maximum barbell back squat of 172.7 ± 25.2 kg. All participants were free of any physical limitations that may have affected performance. Additionally, all participants were free of any prescription or over the counter medications, performance enhancing drugs, and/or ergogenic aids including the use of creatine, beta-alanine or any herbal/vitamin supplement, as determined by a health and activity questionnaire. Following an explanation of all procedures, risks, and benefits, each participant provided his written informed consent prior to participation in this study. The research protocol and the informed consent document were approved by the New England Institutional Review Board prior to participant enrollment (NEIRB# 14-272, Approved 8 August 2014).

### 2.2. Maximal Strength Testing

Prior to experimental trials, participants reported to the Human Performance Laboratory (HPL) to establish maximal strength (1RM) on all lifts involved in the exercise protocol. Participants performed a standardized warm-up consisting of five minutes on a cycle ergometer against a light resistance, 10 body weight squats, 10 body weight walking lunges, 10 dynamic walking hamstring stretches, and 10 dynamic walking quadriceps stretches. Following the warm-up, 1RM testing for the barbell back squat and leg press exercises were performed. Briefly, each participant performed two warm-up sets using a resistance of approximately 40%–60% and 60%–80% of his perceived maximum, respectively. For each exercise, 3–4 subsequent trials were performed to determine the 1RM. A 3–5 min rest period was provided between each trial. Maximum strength testing for the back squat and leg press was administered by the same Certified Strength and Conditioning Specialist (CSCS) to ensure that each participant reached the parallel position for each repetition of the squat and that the exercise technique was consistent between sessions. For the leg press, hamstring curl, and calf raise, 1RM was predicted using the Brzycki prediction equation [18]. Trials were discarded where range of motion criteria for each exercise were not met, where repetitions performed for predicted 1RM were greater than 10, or where proper technique was not used, as determined by the CSCS.

### 2.3. Experimental Trials

On the morning of each trial, participants reported to the HPL after a 10-h overnight fast and having refrained from all forms of moderate to vigorous exercise for the previous 72 h. Experimental trials were performed in a randomized counter-balanced order, and each experimental trial was separated by a minimum of one week to ensure adequate recovery. Each participant performed each trial at the same time of day to avoid diurnal variations. Participants provided a urine sample upon arrival to the HPL for analysis of urine-specific gravity (USG) by refractometry to ensure adequate hydration status (USG < 1.020 defined as euhydration). During each experimental trial, participants performed the standardized warm-up routine as described previously, followed by a lower-body resistance exercise protocol. The resistance exercise protocol required participants to perform 10–12 repetitions with a load of equating to 70% of their 1RM, with a 1-min rest period between each set and exercise. The protocol included six sets of barbell back squats, and four sets of bilateral leg press, bilateral hamstring curls, bilateral leg extensions, and seated calf raises. During each trial, participants were verbally encouraged to complete all repetitions for each set. If the participant was unable to complete the desired number of repetitions, spotters provided assistance until the participant completed the remaining repetitions. Subsequently, the load for the next set was adjusted so that participants were able to perform the specific number of repetitions for each set. Following each resistance exercise protocol, participants remained in the laboratory for all post-exercise assessments. Blood samples were obtained at six time points during each experimental condition: baseline (BL), immediately post-exercise (IP), 30 min post-exercise (30P), 1 h post-exercise (1H), 2 h post-exercise (2H), and 5 h post-exercise (5H). Participants were provided a standardized low protein, low carbohydrate breakfast bar (7 g protein, 3 g carbohydrate and 13 g fat) following BL assessments. The exercise protocol was administered 20 min post-ingestion of the breakfast bar. Immediately following IP blood sampling, participants were provided either a supplement (SUPP) or placebo (PL). Participants were encouraged to consume the beverage within 5 min. The SUPP contained 120 calories, 20 g of milk protein, 6 g of carbohydrates (1 g fiber), and 1 g of fat. The SUPP was independently analyzed for amino acid composition (Eurofins Scientific Inc., Petaluma, CA, USA). The amino acid composition is presented in Table 1. The PL was identical in taste and color, but contained only flavored water (355 mL; 0 g protein, 2.5 g carbohydrates, 0 g fat, 10 calories). Participants were permitted to drink water ad libitum during the experimental trials and post-exercise period, and water consumption was monitored.

### 2.4. Dietary Logs

Participants were instructed to maintain their normal dietary intake leading up to experiment trials. Participants were then instructed to record as accurately as possible everything they consumed during the 24 h prior to the first experimental trial. For the following experimental trial, participants were required to duplicate the content, quantity, and timing of their daily diet during the 24 h prior. Participants were instructed not to eat or drink (except water) within 10 h of reporting to the HPL for experimental trials.

### 2.5. Blood Measurements

During each experimental trial, blood samples were obtained using a Teflon cannula placed in a superficial forearm vein using a three-way stopcock with a male luer lock adapter and plastic syringe. The cannula was maintained patent using a non-heparinized isotonic saline solution (Becton Dickinson, Franklin Lakes, NJ, USA). BL blood samples were obtained following a 15-min equilibration period. IP blood samples were taken within one minute of exercise cessation. Participants were instructed to lie in a supine position for 15 min prior to 30P, 1H, 2H, and 5H blood draws. All blood samples were collected into three 6 mL Vacutainer^®^ tubes (Becton Dickinson, Franklin Lakes, NJ, USA). Blood samples were drawn into either plain, sodium heparin, or K_2_EDTA treated tubes. A small aliquot of whole blood was removed and used for determination of hematocrit and hemoglobin concentrations. The blood in the plain tube was allowed to clot at room temperature for 30 min and subsequently centrifuged at 3000 *g* for 15 min along with the remaining whole blood from the other tubes. The resulting serum and plasma was placed into separate micro-centrifuge tubes and frozen at −80 °C for later analysis.

### 2.6. Biochemical Analysis

Blood lactate concentrations were analyzed from plasma using an automated analyzer (Analox GM7 enzymatic metabolite analyzer, Analox instruments USA, Lunenburg, MA, USA). Hematocrit concentrations were analyzed from whole blood via microcentrifugation (CritSpin, Westwood, MA, USA) and microcapillary technique. Hemoglobin concentrations were analyzed from whole blood using an automated analyzer (HemoCue, Cypress, CA, USA). Plasma volume shifts were calculated using the formula established by Dill & Costill [19]. To eliminate inter-assay variance, all samples were analyzed in duplicate by a single technician. Coefficient of variation for each assay was 1.4% for blood lactate; 0.4% for hematocrit; and 0.6% for hemoglobin.

Plasma concentrations of myoglobin, cortisol (Calbiotech, Spring Valley, CA, USA) and insulin were assessed via enzyme-linked immunosorbent assays (ELISA) and a spectrophotometer (BioTek Eon, Winooski, VT, USA) using commercially available kits. To eliminate inter-assay variance, all samples for each assay were thawed once and analyzed in duplicate in the same assay run by a single technician. Coefficients of variation for each assay were 4.1% for myoglobin, 5.3% for cortisol and 8.1% for insulin.

Plasma samples were assayed for concentrations of monocyte chemoattractant protein 1 (MCP-1) using a multiplex cytokine assay (Milliplex, Cat No. HCYTOMAG-60K; Millipore, Billerica, MA, USA) on a MAGPIX instrument (Luminex, Austin, TX, USA), according to the manufacturer’s instructions. All samples were run in duplicate with a mean intra-assay variance of 8.77%.

### 2.7. Cell Staining

Cell staining was performed as described previously [20]. Analysis of target receptor expression on monocytes was completed at BL, IP, 1H, 2H and 5H time points. K_2_EDTA-treated peripheral whole blood was used to identify monocytes, and quantify target receptor expression by direct immunofluorescence and flow cytometry (BD Biosciences, San Jose, CA, USA). Erythrocytes were first lysed from 350 μL of K_2_EDTA-treated whole blood with BD Pharm Lyse solution (BD Biosciences) within 30 min of collection. Samples were then washed in staining buffer containing Dulbecco’s phosphate-buffered saline (DPBS) with 0.2% (w/v) bovine serum albumin (BSA) (BD Pharmingen Stain Buffer; BD Biosciences) followed by centrifugation and aspiration for a total of three washes. Leukocytes were then resuspended in 100 μL BD Pharmingen stain buffer (BD Biosciences). Direct staining methods were used to label CD14 and CD16 (monocyte identifiers), CCR2 (monocyte chemotaxis) and CD11b (monocyte adhesion). Due to the incorporation of other cell markers not associated with this study, two separate cell preparations were performed.

For cell preparation 1, PerCP Cy5.5 conjugated anti-CD14 (562692; IgG2b; BD Pharmingen™), PE conjugated anti-CD16 (561313; IgG1; BD Pharmingen™), and allophycocyanin (APC) conjugated anti-CD11b (550019; IgG1; BD Pharmingen™), were used in the receptor labeling process. Surface staining for preparation 1 was performed by adding 5 µL of directly conjugated PerCP Cy5.5-anti-CD14, 5 µL of directly conjugated PE-anti-CD16, and 20 µL of directly conjugated APC-anti-CD11b to the cell suspension, followed by incubation in the dark for 30 min at room temperature. Cells were then resuspended in 1.0 mL of stain buffer for immediate flow cytometry analysis.

For cell preparation 2, PerCP Cy5.5 conjugated anti-CD14 (562692; IgG_2b_; BD Pharminigen), and allophycocyanin (APC) conjugated anti-CCR2 (FAB151A; IgG_2b_; R & D Systems, Minneapolis, MN, USA) were used in the receptor labeling process. Surface staining was performed by adding 5 µL of directly conjugated PerCP Cy5.5-anti-CD14, and 10 µL of directly conjugated APC-anti-CCR2 to the cell suspension followed by incubation in the dark for 30 min at room temperature. Cells were then washed in staining buffer followed by centrifugation and aspiration. Cells were subsequently resuspended in 250 µL of fixation and permeabilization solution (BD cytofix/cytoperm™, BD Biosciences), and set to incubate for 20 min in the dark at 4 °C. Following incubation, cells were washed in 1 mL of Perm Wash Buffer (BD Perm/Wash™, BD Biosciences), followed by centrifugation and aspiration, and the addition of 50 µL of Perm Wash Buffer. Cells were then washed in 1.0 mL of Perm Wash Buffer and resuspended in 1.0 mL of stain buffer for flow cytometry analysis following centrifugation and aspiration.

### 2.8. Flow Cytometry

Flow cytometric analysis of stained cells was performed on a BD Accuri C6 flow cytometer (BD Biosciences), equipped with BD Accuri analysis software (BD Biosciences). Forward- and side-scatter, along with four fluorescent channels of data, were collected using two lasers, providing excitation at 488 nm and 640 nm. A minimum of 10,000 events, defined as CD14^+^ monocytes, were obtained with each sample. Compensation for fluorescence spillover was achieved through single staining of anti-mouse Ig, κ/negative control compensation particles (BD CompBeads, BD Biosciences). Unstained leukocytes from human peripheral blood taken at baseline was used as a negative control for CD14, CD16, CCR2 and CD11b expression. Viable cells were obtained using forward-scatter height (FSC-H) × forward-scatter area (FSC-A) gating to eliminate debris, necrotic cells and artifact. The mean fluorescence intensity, which represents the mean density of each receptor per cell, was quantified by overlaying the histogram plots of target receptors to the control samples.

### 2.9. Gating Procedure

Cell preparation 1. Classical monocytes were determined based upon CD14 and CD16 expression as previously described [21]. Analysis of CD11b receptor expression was completed on CD14^++^CD16^−^ cells using one dimensional histograms relative to negative control.

Cell preparation 2. Monocytes were determined via one-dimensional histogram analysis of CD14^+^ cells relative to unstained control. CCR2 is reported to be expressed almost solely on CD14^++^CD16^−^ monocytes [22]. Therefore CD16 was not used for cellular differentiation. Accordingly, CCR2 expression was assessed on CD14^+^ monocytes via one-dimensional histogram analysis relative to unstained control.

### 2.10. Statistical Analysis

Prior to statistical procedures, all data was assessed for sphericity. If the assumption of sphericity was violated, a Greenhouse-Geisser correction was applied. Biochemical and receptor expression changes were analyzed using a two factor (time × treatment) repeated measures analysis of variance (ANOVA). In the event of a significant F ratio, least significant difference (LSD) post-hoc analysis were used for pairwise comparisons. In the event of a significant time effect, follow-up one way repeated measures ANOVA were used to determine time effects for each treatment. If a significant interaction was observed, dependent *t*-tests were used for pairwise comparisons between treatments at each time point. Comparisons between treatments were further analyzed using Cohen’s *d*. Interpretations of Cohen’s *d* were evaluated in accordance with Thalheimer and Cook [23] at the following levels: negligible effect (≥−0.15 and <0.15), small effect (≥0.15 and <0.40), medium/moderate effect (≥0.40 and <0.75), large effect (≥0.75 and <1.10), very large effect (≥1.10 and <1.45), and huge effect ≥1.45). Time effects were further analyzed using partial eta squared (η^2^*_p_*). Interpretations of η^2^*_p_* were evaluated in accordance with Cohen [24] at the following levels: small effect (0.01–0.058), medium effect (0.059–0.137) and large effect (>0.138). The net area under the curve (AUC) was also calculated for biochemical measures using a standard trapezoidal technique, and was assessed using paired samples *t*-tests. Significance was accepted at an alpha level of *p* ≤ 0.05. Data were analyzed using IBM SPSS Statistics for Windows (version 21.0; IBM Corp., Armonk, NY, USA). All data are reported as mean ± SD, unless otherwise stated.

The Shapiro-Wilk test was used to determine normality. The results of this test indicated that cytokine and receptor variables were not normally distributed at a number of time points. Consequently, parametric statistics were supplemented with an analysis based on the magnitude of difference. This analysis does not require the data to be normally distributed. Magnitude-based differences were calculated from 90% confidence intervals, as described previously by Batterham and Hopkins [25]. Differences between BL and all subsequent time points were calculated for each treatment group. This approach uses the smallest worthwhile changes to establish the likelihood (in percentage terms) of the experimental condition having a positive, trivial or negative effect. The change scores were then analyzed via a published spreadsheet [26], with the smallest, nontrivial change set at 20% of the grand SD of all BL values [25]. All data are expressed as a mean effect ± SD, with percent chances of a beneficial, trivial, or negative outcome. Qualitative inferences, based on quantitative chances, were assessed as: <1%, almost certainly not; 1%–5%, very unlikely; 5%–25%, unlikely; 25%–75%, possibly; 75%–95%, likely; 95%–99%, very likely; and 99%, almost certainly [25]. If there was a greater than 5% chance that the true value was both greater and smaller, the effect was considered mechanistically unclear.

## 3. Results

### 3.1. Resistance Exercise Protocol

Exercise volume (sets × load × reps) was similar between PL (53,539 ± 14,178.1 kg) and SUPP (55,027.5 ± 13,048.4 kg) (*d* = 0.12; *p* = 0.405). Changes in plasma lactate concentrations are presented in Table 2. No time × treatment interaction or main effect for treatment (*p* > 0.05) was noted. However, a significant time effect for lactate was observed (F = 169.0; *p* < 0.001; η^2^*_p_* = 0.95). With both trials combined, plasma lactate was significantly elevated above BL at all post-exercise time points (*p*’s < 0.002; Table 2).

### 3.2. Plasma Volume Shifts

No significant time × treatment interaction (*p* = 0.619) or main effect for treatment (*p* = 0.580) was noted between treatments for plasma volume shifts. With both trials combined, plasma volume decreased at IP, −8.8% ± 6.4%; increased at 30P, 0.7% ± 7.6%; increased at 1H, 7.4% ± 12.0%; increased at 2H, 5.0% ± 5.4%; and decreased at 5H, −0.7% ± 5.0% relative to BL. Blood variables were not corrected for plasma volume shifts due to the importance of molar exposure at the tissue receptor level. No significant differences were noted for water consumption between each exercise protocol (*p* = 0.815).

### 3.3. Biochemical Analysis

#### 3.3.1. Myoglobin

Changes in plasma myoglobin concentrations are presented in Table 2. No time × treatment interaction was noted for plasma myoglobin (*p* < 0.05). However, significant main effects for treatment (F = 5.8; *p* = 0.039; η^2^*_p_* = 0.39) and time (F = 29.1; *p* < 0.001; η^2^*_p_* = 0.77) were observed. With both trials combined, plasma myoglobin was significantly elevated at all post-exercise time points (*p*’s < 0.001). AUC analysis revealed no significant differences between treatments (*d* = 0.40; *p* = 0.100).

#### 3.3.2. Cortisol

Changes in plasma cortisol concentrations are presented in Table 2. No significant time × treatment interaction (*p* > 0.863) or main effect for treatment was noted (*p* > 0.665). However, a significant main effect for time was noted for cortisol (F = 29.3; *p* < 0.001; η^2^*_p_* = 0.77). With both trials combined, significant elevations in plasma cortisol concentrations were observed at IP, 30P and 1H (*p* ≤ 0.001), while a significant decrease was noted at 5H relative to BL (*p* = 0.001). AUC analysis revealed no significant differences between treatments for plasma cortisol (*d* = 0.03; *p* = 0.905).

#### 3.3.3. Insulin

Changes in plasma insulin concentrations are presented in Table 2. No significant time × treatment interaction (*p* = 0.722) or main effect for treatment was noted (*p* = 0.560). However, a significant main effect for time was noted for insulin (F = 8.3; *p* < 0.012; η^2^*_p_* = 0.48). With both trials combined, significant elevations in plasma insulin concentrations were observed at 30P (*p* = 0.009) and 1H (*p* = 0.004) relative to BL. A trend was observed towards an increase in plasma insulin at IP relative to BL (*p* = 0.069). AUC analysis revealed no significant difference between treatments for plasma insulin (*d* = 0.14; *p* = 0.228).

### 3.4. Proportion of Monocytes

#### 3.4.1. CD14^+^ Monocytes Relative to All Leukocytes

Changes in percent CD14^+^ cells relative to all leukocytes are presented in Table 3. No time × treatment interaction (*p* = 0.924) or main effect for treatment (*p* = 0.275) was noted. However, a significant time effect was noted for percent CD14^+^ (F = 9.7; *p* = 0.003; η^2^*_p_* = 0.55). With both trials combined, the proportion of cells that were CD14^+^ was significantly decreased at 2H, and 5H, relative to BL (*p*’s < 0.001).

#### 3.4.2. CD14^++^CD16^−^ Monocytes Relative to All Monocytes

Changes in percent CD14^++^CD16^−^ monocytes relative to the entire monocyte pool (classical, non-classical and intermediate) are presented in Table 3. No time × treatment interaction (*p* = 0.318) or main effect for treatment (*p* = 0.616) was noted. However, a significant time effect was noted for percent CD14^++^CD16^−^ monocytes (F = 47.5; *p* < 0.001; η^2^*_p_* = 0.84). With both trials combined, the proportion of monocytes that were CD14^++^CD16^−^ was significantly elevated above BL at 1H, 2H and 5H (*p*’s ≤ 0.001).

### 3.5. Innate Immune Response

#### 3.5.1. MCP-1

Changes in plasma MCP-1 concentrations are presented in Table 4. No time × treatment interaction (*p* = 0.170) or main effect for treatment (*p* = 0.481) was noted. However, a significant time effect was noted for plasma MCP-1 (F = 8.0; *p* = 0.003; η^2^*_p_* = 0.47). With both trials combined, plasma MCP-1 was significantly elevated above BL at all post-exercise time points (*p* < 0.001–0.012). AUC analysis revealed no significant differences between treatments for MCP-1 (*d* = 0.14; *p* = 0.684). Magnitude-based inferences comparing treatment groups can be observed in Table 3. Results indicated that plasma MCP-1 concentrations were “very likely greater” (98.1% likelihood effect) from BL to 2H in the SUPP group compared with PL (*d* = 1.46; mean effect: 186 ± 226 pg·mL^−1^). All other changes in MCP-1 were interpreted as unclear (Table 4).

#### 3.5.2. CCR2

Changes in CCR2 expression are presented in Table 3. A trend towards a time × treatment interaction was noted for CCR2 (F = 2.7; *p* = 0.051; η^2^ = 0.28). However, no significant main effect for treatment (F = 1.7; *p* = 0.239; η^2^ = 0.19) or main effect for time (F = 1.9; *p* = 0.194; η^2^ = 0.22) were observed for CCR2. Magnitude-based inferences revealed that CCR2 expression following SUPP was “likely greater” (84.9% likelihood effect) from BL to IP (mean effect: 770 ± 950 RFU), “likely greater” (87.7% likelihood effect) from BL to 1H (mean effect: 720 ± 780 RFU), “very likely greater” (97.0% likelihood effect) from BL to 2H (mean effect: 1100 ± 830 RFU) and “likely greater” (90.1% likelihood effect) from BL to 5H (mean effect: 1000 ± 1100 RFU) when compared with PL (Table 4).

#### 3.5.3. CD11b

Changes in CD11b expression are presented in Table 3. No significant time × treatment interaction (*p* = 0.501) or main effect for treatment (*p* = 0.122) was noted for CD11b. However, a significant main effect for time was noted (F = 4.7; *p* = 0.004; η^2^*_p_* = 0.35). With both trials combined, CD11b was significantly elevated above BL at 1H post-exercise (*d* = 0.76; *p* = 0.025). A significant decrease in CD11b receptor expression was also observed at 5H post-exercise (*d* = 0.92; *p* = 0.018). All changes in CD11b expression between SUPP and PL over time were interpreted as “unclear” following analysis with magnitude-based inferences (Table 4).

## 4. Discussion

This study examined the effect of an amino acid supplement (SUPP) on plasma MCP-1, monocyte CCR2 expression, and CD11b expression following an acute bout of high-volume, moderate intensity resistance exercise in experienced, resistance-trained men. No differences were noted between treatments for plasma lactate, myoglobin, cortisol, insulin, the proportion of CD14^+^ relative to all leukocytes, or CD14^++^CD16^−^ monocytes relative to all monocytes. Ingestion of SUPP appeared to result in maintained plasma MCP-1 concentrations at 2H post-resistance exercise. SUPP also appeared to augment CCR2 expression on CD14^+^ monocytes at 1H, 2H and 5H post-resistance exercise, although did not appear to have any influence on CD11b expression on CD14^++^CD16^−^ monocytes.

The SUPP utilized in this study was a commercially available chocolate flavored protein supplement, while the PL contained only water with chocolate syrup added for color. Compared to PL, SUPP contained an additional 2.5 g of carbohydrate and 1 g of fat. However, while the SUPP and PL were not isocaloric, it is unlikely that the negligible differences in the FAT and CHO content of SUPP contributed to the observed findings. A recent study by Horvath et al. [27] demonstrated that postprandial plasma MCP-1 concentrations and monocyte CCR2 mRNA are unchanged following infusion of either glucose (20% dextrose solution: blood glucose increased to 220 mg·dL^−1^ over 30 min and sustained for 210 min), lipid (emulsion of 20% soy bean oil, 1.2% egg yolk phospholipids, 2.25% glycerin, and 76.55% water: infusion rate: 1.1 mL·kg·h^−1^) or combination treatment over the course of 4 h in healthy males [27]. Consequently, the differences between SUPP and PL in the present study appear to be related to the amino acid composition of SUPP.

MCP-1 is produced by a number of cells types, including epithelial, mesengial, astrocytic and monocytic cells [28]. Notably, studies indicate that adipocytes and endothelial cells are important sources of MCP-1 [29,30,31]. Adipocytes are reported to exhibit immune cell function with the ability to independently stimulate low-grade inflammation via secretion of MCP-1 [29], while endothelial cells are also reported to increase MCP-1 secretion subsequent to damage [31]. However, these effects are primarily observed in conditions of obesity [32,33] and hypercholesterolemia [34,35]; which, rather than influencing transient changes in the inflammatory response, appear to result in chronically elevated baseline values for MCP-1 and/or CCR2. The cross-over design utilized in the present study negates the potential effect of chronically elevated baseline MCP-1 levels or CCR2 expression. Accordingly, the observed increases in MCP-1 appear to be in response to a transient pro-inflammatory signal. In general, immune responses do not produce endothelial injury [36]. Therefore, given the magnitude of the observed rise in both plasma MCP-1 and myoglobin, along with the exercise stimulus that targeted the lower extremity, it is likely that the MCP-1 response observed is the result of muscle damage. Consistent with our findings, robust elevations in plasma MCP-1 have been previously reported in conjunction with intense exercise [17,37,38]. In agreement with these studies, we have recently reported that MCP-1 is significantly elevated above baseline concentrations for up to 5 h following lower-body resistance exercise [39].

Tissue resident macrophages appear to be the primary source of plasma MCP-1 subsequent to myofibrillar injury [40]. The functional role of resident macrophages in monocyte recruitment is consistent with the dramatic reduction in MCP-1 and monocyte infiltration observed following selective depletion of resident macrophages in injured muscle [3]. Studies utilizing targeted disruption of SCYA2 (the gene encoding MCP-1) provide the physiological basis for MCP-1 involvement in skeletal tissue recovery. These studies demonstrate a delayed restoration of perfusion, and significant decrements in indices of muscle regeneration [7,41], indicating that MCP-1 mediated recruitment of monocytes is essential for recovery. Consistent with this, Deyhle and colleagues [10] recently reported an augmented MCP-1 response following repeated bouts of resistance exercise. Greater intramuscular MCP-1 protein concentrations were associated with an enhanced non-resident macrophage accumulation in damaged tissue, and a subsequent decrease in delayed onset muscle soreness [10]. Deyhle and colleagues [10] appear to have been the first investigative team to link greater concentrations of MCP-1 to enhanced recovery. Our current findings suggest that supplementation with amino acids in healthy males may prolong the MCP-1 response following damaging resistance exercise.

To our knowledge, there is little research examining the effect of amino acids on macrophage derived MCP-1. Therefore, we can only speculate as to a possible mechanism behind our findings. Recent research shows that inhibition of mTOR complex 1 (mTORC1) is associated with a concomitant reduction in MCP-1 secretion by macrophages [12]. Ai and colleagues [12] demonstrated that deletion of regulatory associated protein of mTOR (RAPTOR) results in decreased Ser727 phosphorylation of signal transducer and activator of transcription 3 (STAT3), and a subsequent decrease in STAT3 binding to the MCP-1 promoter. This in turn resulted in a marked decrease in macrophage MCP-1 mRNA expression. This is consistent with previous reports that mTORC1 is responsible for STAT3 phosphorylation [42], and provides a physiological rationale for amino acid modulation of macrophage MCP-1 secretion. Current research does support the mTOR dependent phosphorylation of STAT3 at Ser727 in response to amino acids in vitro [43]. Further, supplementation with essential amino acids has been shown to stimulate mTOR to a greater extent than resistance exercise alone [44]. Nevertheless, the effect of amino acid supplementation on mTOR signaling in macrophages in vivo has not been examined. While it is currently unclear whether the threshold for amino acid induced mTOR activation is conserved between differing cell types, 10 g of essential amino acids has previously been shown to result in a 5.2 fold increase in mTOR activation in skeletal muscle [45]. The amino acid supplement utilized in the present study contained 8.8 g of essential amino acids, which is likely sufficient to potentiate activation of mTOR.

Monocyte chemotactic function is mediated via exclusive binding of MCP-1 to CCR2 [46]. We observed a strong trend (*p* = 0.051) towards an interaction between treatments. Further analysis with magnitude-based inferences indicated that CCR2 expression was augmented following ingestion of SUPP at 1H, 2H and 5H post-exercise. These findings were somewhat unexpected. Upon encountering inflammatory stress signals, classical monocytes must rapidly activate and migrate to areas of injury. This requirement dictates that cell receptors be both present, and expressed in adequate number in anticipation of environmental stimuli. A recent study examining resting chemokine receptor expressions in healthy individuals demonstrated that CCR2 is expressed at a high frequency (93.1%), and a high median fluorescence intensity on classical monocytes [22]. Additionally, the frequency and expression of CCR2 was stable over the course of a three-day analysis, suggesting that CCR2 is constitutively expressed on classical monocytes at a high level. Accordingly, it seems unlikely that CCR2 would undergo an up-regulation in expression in response to a nutritional stimulus. We have recently shown that CCR2 undergoes a down-regulation following resistance exercise [39], which is likely due to receptor cycling following interaction with MCP-1 [47]. Our current data seem to support these findings. However, ingestion of SUPP appears to have attenuated the decline in CCR2 receptor expression following exercise. Further, these differences do not appear to be related to MCP-1 modulation of receptor cycling, since MCP-1 concentrations were also maintained in SUPP. There is currently no evidence to suggest that mTOR regulates CCR2 expression in monocytes. However, a recent study demonstrated that inhibition of the alpha 1 subunit of adenosine monophosphate-activated protein kinase (AMPKα1) in the murine RAW264.7 macrophage cell line, led to increased CCR2 expression in both resting and activated (M1) states [48]. Further, pharmacological activation of AMPK was shown to inhibit CCR2. Previous research demonstrates that AMPK signaling negatively controls mTOR signaling [49]. Further the same study demonstrated that l-leucine stimulates mTOR activation, in part through AMPK inhibition [49]. Together these data provide a potential mechanism for amino acid regulation of CCR2 expression in monocytes. Additional research is needed to substantiate this mechanism.

Interaction of MCP-1 with CCR2 is reported to induce rapid internalization of the MCP-1/CCR2 receptor complex [47]. Following internalization, MCP-1 disassociates from the CCR2 receptor and is transported to the lysosome, where it is subsequently degraded [47]. Upon dissociation, CCR2 rapidly cycles back to the cell surface in order to maintain the responsiveness of the cell towards the chemokine. Under these conditions, MCP-1 is being continuously removed from the systemic environment. Consequently, it is possible that additional MCP-1 must be secreted in order to maintain the necessary chemotactic gradient for optimal monocyte responsiveness. It is feasible that the augmentation of CCR2 receptor expression observed in the present study, particularly at 2H, may have resulted in additional MCP-1 being removed from the systemic environment. Although speculative, this in turn may have triggered an increase in MCP-1 secretion for the purpose of maintaining monocyte responsiveness.

CD11b mediates the cellular adhesion and intravascular crawling of monocytes, which is the direct pre-requisite to tissue infiltration [50]. CD11b has previously been shown to undergo up-regulation in response to resistance exercise [39]. The up-regulation of CD11b could potentiate a greater capacity for monocyte adhesion at sites of tissue damage, which may result in modified muscle recovery. Consistent with these studies, CD11b expression was up-regulated at 1H post-exercise. However, no time × treatment interaction was observed. Further, differences between treatments were interpreted as unclear at all time-points. Consequently, our results indicate that ingestion of SUPP did not affect CD11b expression on CD14^++^CD16^−^ monocytes. To our knowledge, only one other study has examined changes in CD11b expression following a nutritional intervention. Gonzalez and colleagues [51] recently examined changes in CD11b expression on CD14^+^ monocytes following supplementation with the free-acid form of the leucine metabolite, beta-hydroxy-beta-methylbutyrate (HMB). Although no time effects were noted in the HMB group, HMB appeared to attenuate the rise in CD11b observed following ingestion of placebo. These findings suggest that HMB could blunt monocyte infiltration to damaged tissue. Notwithstanding, the percentage of monocytes expressing CD11b was significantly elevated for up to 48 h following HMB supplementation. Theoretically, the increased number of monocytes expressing CD11b could potentially ameliorate any deficits in monocyte adhesion resulting from the observed down regulation of CD11b expression. The implications of these contrasting inflammatory responses are not clear. Nevertheless, it does suggest that CD11b expression is subject to regulation by nutritional stimuli. Additional research is needed to further establish the effects of amino acids and/or their metabolites on CD11b expression.

It is becoming increasingly clear that amino acids, particularly branched chain amino acids, are important regulators of immune responses [52]. These results provide new evidence to suggest that amino acids may modulate plasma MCP-1 concentrations, possibly through interacting with tissue resident macrophages, while augmenting CCR2 expression on CD14^+^ monocytes. Nevertheless, these results should be interpreted with caution, since the effects of the subsequent inflammatory events are yet to be determined. Further, the optimal concentration of plasma MCP-1 for muscle recovery and the optimal magnitude of monocyte infiltration remain unclear. Future research should consider examining mTOR signaling in tissue resident macrophages in conjunction with damaging resistance exercise and oral amino acid administration. Monocyte infiltration in response to differing MCP-1 concentrations, and their subsequent effect on indices of recovery also needs to be addressed, as does the potential for amino acid modulation of CCR2 expression.

In summary, the present study examined the effect of an orally administered amino acid supplement on markers of monocyte recruitment following an acute bout of high-volume, moderate intensity resistance exercise in experienced, trained men. No differences between treatment groups were observed for the myoglobin, cortisol or insulin response to exercise. SUPP ingestion appeared to maintain plasma MCP-1 concentrations through 2H post-resistance exercise and augment CCR2 expression at 1H, 2H and 5H, but did not appear to influence CD11b expression on CD14^++^CD16^−^ monocytes.

## Figures and Tables

**Table 1:** Amino acid composition of SUPP.

**Essential Amino Acids (g/Serving)**	
Leucine	1.86
Isoleucine	0.98
Valine	1.2
Histidine	0.53
Methionine	0.53
Phenylalanine	0.96
Threonine	0.84
Tryptophan	0.29
Lysine	1.59
**Total**	8.78
**Nonessential Amino Acids (g/Serving)**	
Serine	1.08
Glutamic acid	4.07
Proline	1.85
Glycine	0.36
Alanine	0.67
Aspartic acid	1.51
Tyrosine	0.95
Arginine	0.72
Cystine	0.13
**Total**	11.34

SUPP = supplement; g = grams.

**Table 2:** Biochemical response to intervention.

Marker	Trial	BL	IP	30P	1H	2H	5H
Lactate (mmol)	PL	1.4 ± 0.4	12.6 ± 2.5 ^‡^	7.0 ± 1.8 ^‡^	3.9 ± 1.0 ^‡^	2.4 ± 1.1 ^‡^	2.7 ± 1.4 ^‡^
	SUPP	1.5 ± 0.6	12.0 ± 2.3 ^‡^	6.8 ± 1.6 ^‡^	4.2 ± 1.0 ^‡^	2.3 ± 0.7 ^‡^	2.9 ± 1.3 ^‡^
Myoglobin (ng·mL^−1^)	PL	35.0 ± 13.4	91.9 ± 26.1 ^‡^	104.9 ± 34.5 ^‡^	141.9 ± 51.0 ^‡^	141.1 ± 53.6 ^‡^	92.5 ± 32.1 ^‡^
	SUPP	24.1 ± 5.6	77.8 ± 31.7 ^‡^	86.6 ± 27.4 ^‡^	133.4 ± 65.1 ^‡^	124.0 ± 67.4 ^‡^	76.0 ± 32.3 ^‡^
Cortisol (nmol/L)	PL	550.5 ± 293.1	1074.3 ± 474.5 ^‡^	1166.8 ± 393.0 ^‡^	995.2 ± 325.2 ^‡^	622.8 ± 220.6	288.0 ± 221.1 ^§^
	SUPP	580.2 ± 221.9	995.1 ± 498.5 ^‡^	1218.2 ± 430.7 ^‡^	893.4 ± 272.6 ^‡^	619.5 ± 269.2	328.3 ± 192.8 ^§^
Insulin (µIU·mL^−1^)	PL	5.11 ± 3.23	8.28 ± 7.94	15.63 ± 17.58 ^‡^	5.68 ± 2.05 ^‡^	5.04 ± 2.36	4.34 ± 1.16
	SUPP	4.51 ± 1.22	7.34 ± 5.28	16.87 ± 9.92 ^‡^	8.67 ± 3.80 ^‡^	5.31 ± 2.62	4.34 ± 1.45

Treatment groups: PL = Placebo; SUPP = Supplement. Time points: BL = Baseline; IP = Immediately-post; 30P = 30 min post; 1H = One hour post; 2H = Two hours post; 5H = Five hours post. ^‡^ = Significant increase relative to BL (*p* ≤ 0.05); ^§^ = Significant decrease relative to BL (*p* ≤ 0.05); Data reported as means ± SD.

**Table 3:** Changes in percent proportion of CD14^+^ monocytes relative to all leukocytes and CD14^++^CD16^−^ classical monocytes relative to all monocytes.

Variable	Trial	BL	IP	1H	2H	5H
% CD14^+^	PL	11.6 ± 2.7	10.2 ± 3.3	9.7 ± 2.8	8.4 ± 2.0 ^§^	7.7 ± 1.6 ^§^
	SUPP	10.5 ± 3.1	9.2 ± 2.5	9.2 ± 2.9	7.1 ± 1.6 ^§^	7.5 ± 2.1 ^§^
% CD14^++^CD16^−^	PL	86.6 ± 4.5	84.5 ± 5.7	96.4 ± 1.3 ^‡^	94.9 ± 3.1 ^‡^	91.8 ± 2.1 ^‡^
	SUPP	84.6 ± 6.1	85.1 ± 5.6	96.0 ± 2.0 ^‡^	95.7 ± 0.8 ^‡^	90.1 ± 4.9 ^‡^

Treatment groups: PL = Placebo; SUPP = Supplement. % CD14^+^ = percent monocytes relative to all leukocytes; % CD14^++^CD16^−^ = percent classical monocyte relative to all monocytes; Time points: BL = Baseline; IP = Immediately-post; 30P = 30 min post; 1H = One hour post; 2H = Two hours post; 5H = Five hours post. ^‡^ = Significant increase relative to BL (*p* ≤ 0.05); ^§^ = Significant decrease relative to BL (*p* ≤ 0.05); Data reported as means ± SD.

**Table 4:** Magnitude-based inferences comparing cytokine, receptor expression, and proportion of monocytes in response to PL and SUPP.

Variable	Mean Difference		Effect Size	Percent			Interpretation
	PL	SUPP	*d*	Inc	Triv	Dec	
**MCP-1**							
BL vs. IP	245 ± 231	179 ± 152	0.36	25.3	6.1	68.5	Unclear
BL vs. 30P	354 ± 336	190 ± 142	0.67	7.7	2.7	89.7	Unclear
BL vs. 1H	351 ± 295	337 ± 365	0.04	41.5	7.2	51.3	Unclear
BL vs. 2H	44 ± 69	230 ± 177	1.46	98.1	0.8	1.1	Very Likely Greater
BL vs. 5H	116 ± 194	132 ± 175	0.09	52.3	9.3	38.4	Unclear
**CCR2**							
BL vs. IP	−848 ± 1256	−82 ± 215	0.82	84.9	10.2	4.9	Likely Greater
BL vs. 1H	−875 ± 1074	−152 ± 182	1.10	87.7	9.4	2.9	Likely Greater
BL vs. 2H	−959 ± 1275	191 ± 1126	1.01	97.0	2.4	0.6	Very likely Greater
BL vs. 5H	−956 ± 1373	71 ± 293	1.09	90.1	6.4	3.5	Likely Greater
**CD11b**							
BL vs. IP	2101 ± 4705	2 ± 2782	0.57	6.8	15.8	77.3	Unclear
BL vs. 1H	4103 ± 4210	1642 ± 4941	0.57	6.9	13.3	79.8	Unclear
BL vs. 2H	603 ± 5042	−626 ± 5662	0.24	12.3	25.4	62.3	Unclear
BL vs. 5H	−2134 ± 3298	−2181 ± 2645	0.02	26.3	44.9	28.8	Unclear

Groups: PL = Placebo; SUPP = Supplement; MCP-1 = monocyte chemoattractant protein 1; CCR2 = C-C chemokine receptor 2; CD11b = macrophage-1 antigen (cluster of differentiation 11b); Time points: BL = Baseline; IP = Immediately-post; 30P = 30 min post; 1H = One hour post; 2H = Two hours post; 5H = Five hours post; *d* = Cohen’s *d*, Inc = Increase, Triv = Trivial, Dec = Decrease; Data reported as mean ± SD.
